# Jasmonate Signalling Contributes to Primary Root Inhibition Upon Oxygen Deficiency in *Arabidopsis thaliana*

**DOI:** 10.3390/plants9081046

**Published:** 2020-08-17

**Authors:** Vinay Shukla, Lara Lombardi, Ales Pencik, Ondrej Novak, Daan A. Weits, Elena Loreti, Pierdomenico Perata, Beatrice Giuntoli, Francesco Licausi

**Affiliations:** 1 Plantlab, Institute of Life Sciences, Scuola Superiore Sant’Anna, 56127 Pisa, Italy; vinay.shukla@unige.ch (V.S.); d.weits@santannapisa.it (D.A.W.); p.perata@santannapisa.it (P.P.); beatrice.giuntoli@unipi.it (B.G.); 2 Department of Biology, University of Pisa, 56126 Pisa, Italy; lara.lombardi@unipi.it; 3 Laboratory of Growth Regulators, Faculty of Science, Palacký University & Institute of Experimental Botany, The Czech Academy of Sciences, CZ-783 71 Olomouc, Czech Republic; alespencik@seznam.cz (A.P.); ondrej.novak@upol.cz (O.N.); 4 The Institute of Agricultural Biology and Biotechnology, National Research Council, 20133 Milan, Italy; loreti@ibba.cnr.it; 5 Department of Plant Sciences, University of Oxford, Oxford OX1 3RB, UK

**Keywords:** root hypoxia, oxygen sensing, jasmonate, root meristem

## Abstract

Plants, including most crops, are intolerant to waterlogging, a stressful condition that limits the oxygen available for roots, thereby inhibiting their growth and functionality. Whether root growth inhibition represents a preventive measure to save energy or is rather a consequence of reduced metabolic rates has yet to be elucidated. In the present study, we gathered evidence for hypoxic repression of root meristem regulators that leads to root growth inhibition. We also explored the contribution of the hormone jasmonic acid (JA) to this process in *Arabidopsis thaliana*. Analysis of transcriptomic profiles, visualisation of fluorescent reporters and direct hormone quantification confirmed the activation of JA signalling under hypoxia in the roots. Further, root growth assessment in JA-related mutants in aerobic and anaerobic conditions indicated that JA signalling components contribute to active root inhibition under hypoxia. Finally, we show that the oxygen-sensing transcription factor (TF) RAP2.12 can directly induce Jasmonate Zinc-finger proteins (JAZs), repressors of JA signalling, to establish feedback inhibition. In summary, our study sheds new light on active root growth restriction under hypoxic conditions and on the involvement of the JA hormone in this process and its cross talk with the oxygen sensing machinery of higher plants.

## 1. Introduction

Heavy soil structure and intense rainfalls often result in waterlogging events that impose severe hypoxic conditions to the root system of plants due to the poor diffusivity of gases in water [1]. Establishment of anaerobic conditions triggers an energy crisis in root cells due to the fact that oxygen is required by the mitochondrial electron transport chain as a terminal acceptor. Indeed, plant tissues that experience hypoxia mainly rely on glycolysis supported by fermentation for ATP production, although this is considerably lower than that obtained via oxidative phosphorylation [2]. Additionally, restricted oxygen supply affects a wide variety of biochemical processes in which this molecule participates as a substrate, including the production of reactive oxygen species (ROS), fatty acid desaturation and synthesis of phytohormones [3,4]. Therefore, plants developed an array of strategies to save energy and to selectively dedicate the little amount produced to structural maintenance and stress endurance [5].

In higher plants, several of these metabolic adaptations are transcriptionally controlled by constitutively expressed members of the group VII of Ethylene Response Factors (ERF-VII) [6,7,8]. For example, the Arabidopsis ERF-VII RAP2.12 was shown to re-localise from the plasma membrane into the nucleus to induce the expression of enzymes required for fermentative pathways in response to a drop in oxygen availability [9,10]. Indeed, oxygen levels determine ERF-VII stability by acting upon the amino terminal sequence of these transcriptional regulators and by addressing them to proteolysis via the N-end rule pathway [11,12]. This regulatory function has been demonstrated to be catalysed by plant cysteine oxidases, enzymes that use molecular oxygen as a substrate to oxidize an exposed cysteine residue to its sulfinated or possibly sulfonated form [13,14,15]. N-terminal oxidized cysteines are subjected to the subsequent activity of Arginyl-transferases (ATEs) that conjugate an arginine, thereby marking the protein for recognition by the E3 ubiquitin ligase Proteolysis 6 (PRT6) and subsequent proteasomal degradation [16]. Under hypoxia, this chain of events is inhibited at the initial oxidation step, leading to ERF-VII accumulation in the nucleus, where they reprogram the cell transcriptome to accommodate anaerobic metabolism [17,18].

Notwithstanding these metabolic adjustments, growth and developmental penalties are evident in plants that face waterlogging stress [19]. In soybean roots, rhizosphere hypoxia has been shown to mimic the morphological responses caused by waterlogging [20], indicating that the low oxygen component of this stress condition holds major potential to direct root architectural variation [21,22,23]. In general, adaptation to abiotic stresses in roots is guided by genetically controlled post-embryonic root development that allows phenotypic plasticity, fundamentally by the determination of cell division in the apical and lateral root meristems and cell expansion in the elongation zone [24,25,26]. Anatomical root adaptation to hypoxia is species specific. In Rumex and tomato, for example, it involves a reduction in root extension, accompanied by divergence from its regular gravitropic habitus and inhibition of lateral root production in favour of above-water adventitious roots [27,28]. Additionally, some species, including rice, are shown to develop aerenchyma and barriers to radial oxygen losses to enhance oxygen channelling from above-water tissues to the submerged organs [29,30].

Previously, microarray studies have been carried out to investigate the reprogramming of gene expression and metabolism in Arabidopsis roots and to identify a highly specific set of genes induced at moderate levels of hypoxia [31]. Cell-specific genetic regulation across individual cell types of Arabidopsis roots under hypoxia has been studied further at the translatome level [32]. Crosstalk of hypoxia responsive factors and hormonal signalling has been described for ethylene, gibberellic acid and abscisic acid signalling pathways. Ethylene, accumulated under submergence, plays a crucial role in the adaptation to low oxygen environment as well as developmental control in concert with gibberellic acid (GA) and abscisic acid (ABA) signalling. Ethylene promotes shoot elongation in an escape strategy of submergence tolerance in rice [33,34]. The interplay of ethylene with GA and ABA is known to control adventitious root and aerenchyma formation in rice [35,36]. In tomato, the ethylene and auxin signalling pathways crosstalk to ensure adventitious root formation in flooded tomato plants [24]. Instead, the impact of hypoxia on jasmonic acid (JA) signalling pathways is yet to be studied in depth.

JA plays crucial roles in both plant defence and developmental processes [37], including root growth [38], tuberization, tendril coiling [39], senescence [40] and fertility [41]. The contribution of JA to root development has been object of several studies that shed light on a dual role for this hormone. In fact, JA and auxin signalling modules are connected by the transcription factors MYC2 [42] and PLETHORA (PLT1 and PLT2), crucial regulators of root meristem activity and stem cell maintenance depending on auxin gradients. MYC2 has been shown to repress PLT1/2 expression by directly binding to their promoters [43], de facto counteracting the positive contribution to auxin biosynthesis by JA, which occurs via the enzymatic step catalysed by Anthranilate Synthase A1 (ASA1) [44].

In the current study, we investigated the crosstalk of hypoxia on JA signalling in the root apical meristem to test whether root growth was hindered by oxygen deficiency not only as a consequence of reduced metabolic activity but also due to active repressive signalling.

## 2. Results

### 2.1. Primary Root Growth Is Transiently Inhibited under Hypoxia

**Figure 1:**
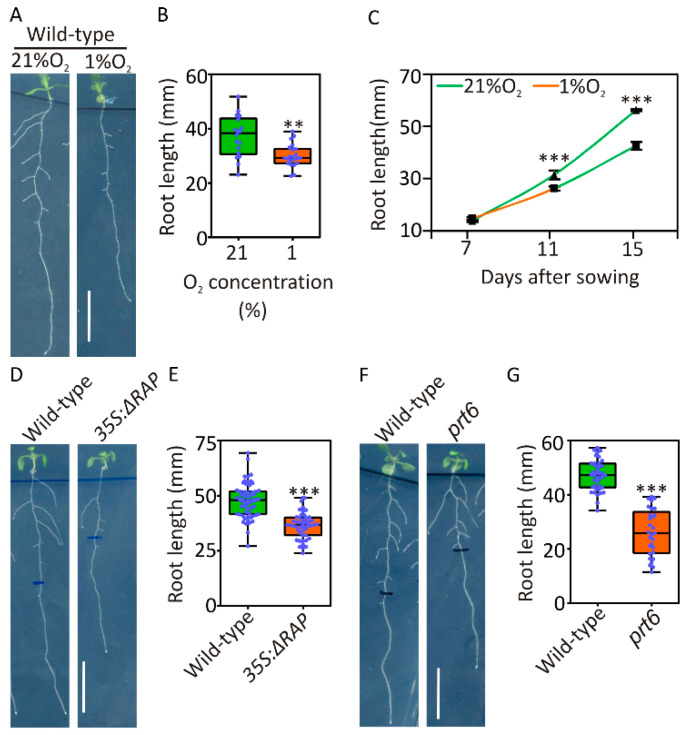
Root length restriction by hypoxia and ERF-VII stabilisation: (**A**) Root development of 7-day-old plants treated with 1% or 21% *v*/*v* O_2_ for 4 days; (**B**) primary root length of 7-day-old plants treated with 1% or 21% *v*/*v* O_2_ for four days (*n* ≥ 20); and (**C**) primary root length of plants grown on vertical plates 7, 11 and 15 days after sowing. Circles represent averages of plants subjected to 4-day-long hypoxia (1% O_2_ *v*/*v*) and subsequently to 4 days of recovery. Error bars represent the standard deviation of *n* ≥ 20. (**D**) Root phenotype of 11-day-old Col-0 and Δ13RAP plants under normoxic conditions; (**E**) root length of wildtype and Δ13RAP plants after 11 days of growth on vertical plates under normoxic conditions (*n* ≥ 40); (**F**) root phenotype of wildtype and prt6 mutant plants after 11 days of growth on vertical plates under normoxic conditions; and (**G**) root length of wildtype and prt6 plants after 11 days of growth on vertical plates under normoxic conditions (*n* ≥ 25): Asterisks indicate statistical significance (** *p*-value ≤ 0.01, *** *p*-value ≤ 0.001, t-test).

To examine the extent to which reduced oxygen levels affect root growth, we compared the primary root length of Arabidopsis seedlings treated with either normoxic (21% O_2_) or hypoxic conditions (1% *v*/*v* O_2_) for four days after one week of aerobic growth. Plants were supplemented with 1% sucrose to prevent possible carbon starvation. As expected and reported previously [28], the length of the primary root was significantly reduced in plants subjected to a hypoxic atmosphere (Figure 1A,B), raising the question of whether this phenomenon was due to transient inactivation of root meristem or rather due to stem cell death. To test these alternative hypotheses, we repeated the experiment, this time including root length measurements also at the onset of hypoxic stress and four days after its end. We observed that primary roots actually maintained elongation under hypoxia and grew further in the post-hypoxia phase, although to a significantly reduced rate when compared to the controls maintained under constant aerobiosis (Figure 1C), indicating that the meristematic activity at the root apex was not lost. Remarkably, root growth inhibition was maintained in the post-hypoxic phase (Figure 1C, Supplementary Figure S1), suggesting that active repression of root growth was established by hypoxia rather than by being a simple metabolic consequence of reduced respiratory metabolism.

Indeed, reduction of root growth under hypoxic conditions can be explained as a consequence of ATP shortage although one could also speculate that repressive signalling may be set in action to save energy and resources and possibly to reorient root growth towards areas with more favourable oxygen conditions. Therefore, we tested whether the regulators of the molecular response to anaerobiosis, the ERF-VII transcription factors [8], may also play a role in regulating root growth under hypoxic conditions. We compared root growth of wildtype and transgenic genotypes in which ERF-VII signalling is constitutively active due to constitutive stabilisation of these TFs independently of the actual oxygen availability. More specifically, we exploited the ΔRAP line that expresses an N-terminally deleted version of the ERF-VII RAP2.12 that makes it insensitive to oxygen [18] and the *prt6* mutant, which bears a nonfunctional version of the E3 ligase responsible for recognition and ubiquitination of ERF-VII proteins [16]. At the transcriptional level, both genotypes exhibit an active anaerobic response even when their mitochondria are perfectly able to carry out oxidative phosphorylation [17]. After 11 days of growth on vertical agar plates, we noticed that the primary root length of the ΔRAP line was significantly reduced when compared to Col-0 plants (Figure 1D,E). Similarly, we observed reduced root growth in the prt6 mutant as compared to wildtype plants (Figure 1F,G). Since hypoxia and ERF-VII stabilisation under aerobic conditions lead to inhibition of primary root growth to a similar extent, we hypothesized that the observed phenotype is at least partly ascribable to active signalling rather than exclusive metabolic arrest.

### 2.2. Root Meristem Regulators Are Repressed by Hypoxia

**Figure 2:**
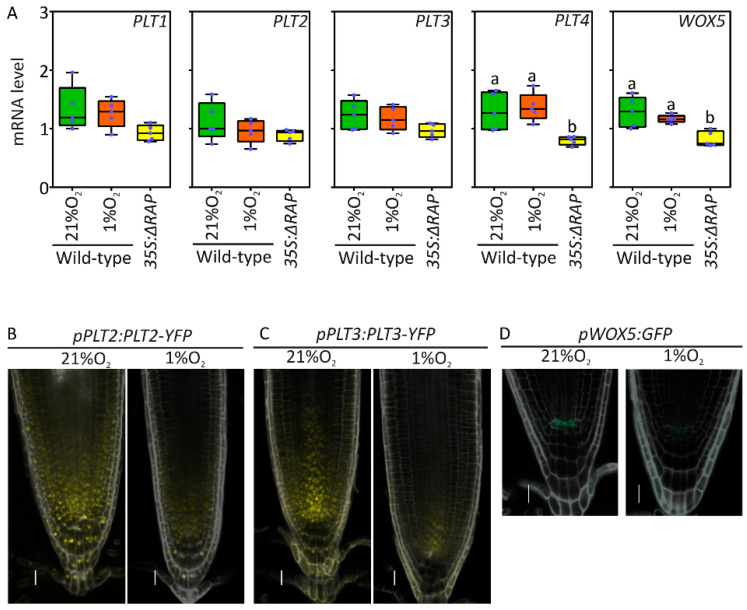
ERF-VII-dependent regulation of transcription factors involved in meristem activity: (**A**) Expression profiles of PLT1-4 and WOX5 in wildtype roots under aerobic and hypoxic conditions and in 35S:∆RAP plants grown in normoxia. mRNA level of each gene is normalised to the house-keeping gene (UBQ10-AT4G05320). Data shown in the graphs are the re-normalised ΔΔCt values to one of the biological replicates of the wildtype sample at 21% O_2_. The letters indicate statistically significant difference calculated by 1-way ANOVA and Tukey post hoc test (*p* ≤ 0.05). (**B-C**)The YFP signal in the root apex of pPLT2:PLT2-YFP (**B**) and pPLT3:PLT3-YFP (**C**) plants maintained 12 h under aerobic (21% O_2_) and hypoxic (1% O_2_) conditions (**D**) GFP signal in the quiescent centre (QC) of pWOX5:GFP plants treated 12 h under normoxia (21% O_2_) or hypoxia. (1% O_2_). The yellow colour is associated with YFP signal, the green colour is assigned to the GFP signal, while grey is used for propidium iodide staining of or cell walls (Scale bar = 20 μm).

Root elongation is ensured by the production of new cells at the apex. Here, basally channelled auxin and apically diffused (CLAVATA3/Embryo Surrounding Region-Related) (CLE)-like peptides restrict the expression of the WUSCHEL-related homeobox 5 (WOX5) transcriptional regulator to the quiescent centre (QC), which in turn promotes cell proliferation in the surrounding meristematic neighbours in a cell nonautonomous manner through the PLETHORA (PLT) family [45]. mRNA quantification of PLTs and WOX5 gene expression in root apex samples did not reveal significant alterations in response to hypoxia, although PLT4 and WOX5 expression was significantly reduced in ΔRAP plants as compared with wildtype ones (Figure 2A). Instead, in PLT2 and PLT3, accumulation was reduced in the meristem as a consequence of 12 h of exposure to 1% oxygen, as revealed by the respective pPLT:PLT-YFP translational reporter lines [44] (Figure 2B,C, Supplementary Figure S2). Similarly, GFP expression was strongly inhibited in the QC when its coding sequence was placed under control of the WOX5 promoter (Figure 2D). We therefore speculated that hypoxia drove posttranscriptional repression of PLT2 and 3 expression, whereas low oxygen-induced stabilisation of RAP2.12 caused WOX5 transcriptional inhibition in the QC. Since the GFP reporter is controlled by the whole genomic region upstream of WOX5 coding sequence, posttranscriptional regulation impacting on the 5′UTR could also not be excluded. The overall reduction in transcripts and proteins involved in root meristem activity under hypoxia can potentially explain the reduction in root growth observed in the previous experiments (Figure 1A).

### 2.3. Assessment of Jasmonate Levels and Activity in Hypoxic Roots

**Figure 3:**
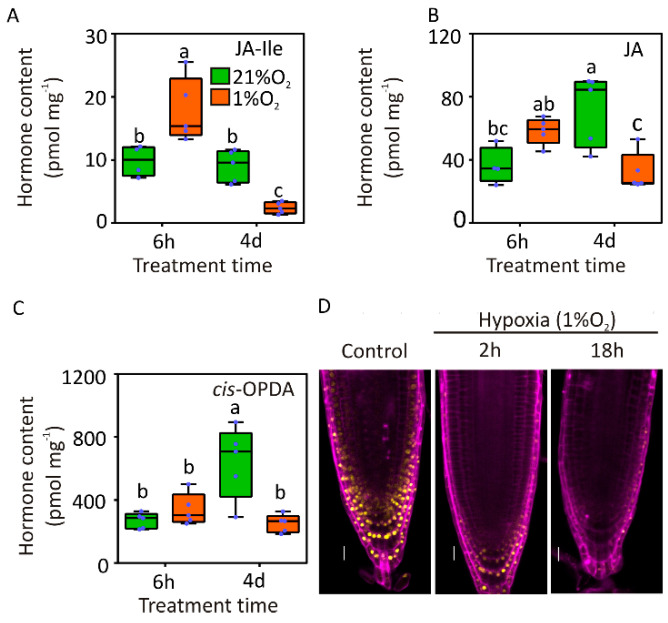
Active jasmonates are produced in roots under hypoxia. Levels of JA (**A**), JA-Ile (**B**) and cis-12-oxo-phytodienoic acid (cis-OPDA) (**C**) in the roots of Arabidopsis plants kept at 21% O_2_ (green boxes) or 1% O_2_ (red boxes) (*n* = 4). The letters in A-C indicate statistical significance compared with untreated controls (*p* ≤ 0.05, Two-way ANOVA, Tukey’s post hoc test). (**D**) Jas9-VENUS fluorescent protein abundance in root apical meristems under aerobic and hypoxic conditions: Yellow associates to the YFP signal, and purple associates to propidium iodide dye (Scale bar = 20 μm).

Primary root growth inhibition was one of the first physiological effects attributed to JA [46]. Therefore, we decided to investigate whether hypoxia could impact JA signalling in roots. First, we quantified the levels of free JA, the bio-active JA-isoleucine conjugate (JA-Ile) [47] and the JA-precursor cis-12-oxo-phytodienoic acid (cis-OPDA) [48] in roots of wildtype plants grown under aerobic and hypoxic conditions. We sampled the roots at the end of two hypoxia treatments, a short period of 6 h and a prolonged one of 4 d, both starting after seven days of normoxic growth. After 6 h hypoxia, we could not observe statistically significant changes in endogenous JA and cis-OPDA levels while the active form JA-Ile was increased (Figure 3A–C). Prolonged exposure to hypoxia, instead, led to significant reduction in JA, JA-Ile and cis-OPDA when compared to aerobic roots (Figure 3A–C). This latter observation is not unexpected, since JA biosynthesis involves several biochemical steps that use oxygen as a co-substrate, including cycles of beta-oxidation in the peroxisomes [49].

To understand whether the observed increase in active JA levels in Arabidopsis roots subjected to hypoxia actually impact on JA signalling, we exploited a genetically encoded reporter for JA activity in plants, consisting of the JA-labile JAS domain of the JAZ9 protein fused to the VENUS yellow fluorescent protein, of which degradation relates directly to the amount of active JA [50]. Indeed, treatment with 1% O_2_, a level of hypoxia that we previously proved to be able to promote a physiological response in roots (Figure 1A), decreased the fluorescent signal in the entire meristematic region (Figure 3D). The signal was decreased as early as 2 h after the onset of hypoxia and did not reappear in the following 16 h (Figure 3D), confirming JA signalling being activated under low oxygen conditions.

### 2.4. JA-Responsive Genes Are Affected in Hypoxic Roots

**Figure 4:**
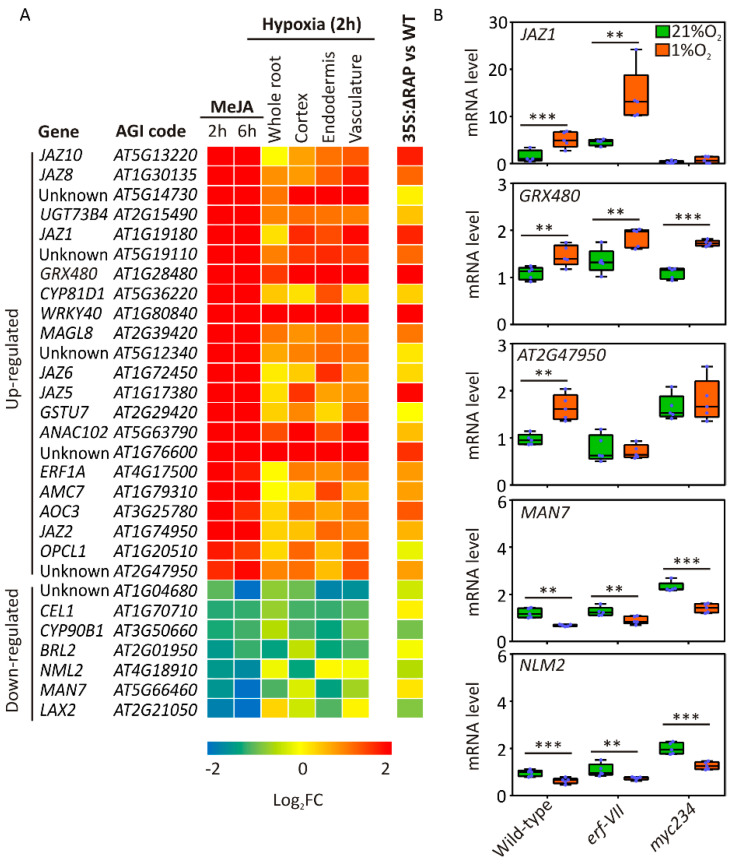
Jasmonic acid (JA)-responsive genes are affected by hypoxia. (**A**) Comparative expression profiles of highly MeJA-responsive genes [51] across root cell types kept in a hypoxic environment [32] or in a ΔRAP2.12 leaf dataset: the colours depict relative expression in each condition, with red indicating upregulation and blue indicating downregulation according to a logarithmic scale. (**B**) Expression of five selected hypoxia and MeJA-responsive genes in wildtype, erf-VII and myc234 mutants under aerobic and hypoxic conditions: mRNA level of each gene is normalised to the house-keeping gene (UBQ10-AT4G05320). Data shown in the graphs are the re-normalised ∆∆Ct values to one of the biological replicates of the wildtype sample at 21% O_2_. Data are mean ± SD of five biological replicates. Asterisks mark statistically significant differences in the indicated pairwise comparisons (**p ≤ 0.01, *** *p* ≤ 0.001, t- test).

In order to understand the contribution of JA signalling to the hypoxic response in Arabidopsis, we first analysed a microarray dataset that interrogates the transcriptional effect of exogenous methyl-JA (MeJA) treatment on cultured Arabidopsis cells for 2 and 6 h [51]. Using the Genevestigator software [52], we selected 70 genes that exhibited the strongest up- (43) or downregulation (27) in response to Me-JA treatments, and associated their corresponding fold change in a hypoxic dataset where cell-specific translatomes from aerobic and hypoxic root samples were compared (Supplementary Table S2) [32]. This analysis returned 22 hypoxia-inducible hormone-responsive mRNAs, characterized by a hypoxic fold change (FC) larger than 2 and 7 in which abundance was at least halved in one root cell type (Figure 4A, Supplementary Table S3). Among the upregulated genes, six coded for JAZ proteins (JAZ1/2/5/6/8/10), established markers of JA responses. Analysing the impact of RAP2.12 stabilisation under aerobic conditions [18] on the same set of genes, we observed a remarkable overlap: 9 of the 11 upregulated genes were induced, and 8 of the 13 downregulated ones were repressed by RAP2.12 (Figure 4A). This last observation indicated the capacity of ERF-VII TFs to regulate JA-responsive genes, either, in principle, by direct transcriptional control or rather by promotion of active JA signalling. In the first instance, we identified candidate ERF-VII target genes in those containing the Hypoxia Responsive Promoter Element (HRPE), bound by ERF-VII proteins [9], in the 1 kb 5′ region upstream of their coding sequence. Since we could detect this DNA motif in 3 of the 24 selected genes only (Supplementary Table S3), we rather favoured the hypothesis of indirect regulation exerted by these transcription factors on JA-responsive genes.

Among the 11 upregulated genes, we identified JAZ1 (jasmonate-ZIM Domain 1), a repressor of JA signalling [53]. An in-depth analysis of the whole JAZ gene family revealed that also JAZ8 is induced by constitutive expression of the oxygen-insensitive RAP2.12 variant (Supplementary Figure S3).

To demonstrate the involvement of the ERF-VII-mediated oxygen sensing pathway, on one side, and of the MYC-dependent jasmonate signalling, on the other, we analysed the root tissues of wildtype, erf-VII and myc234 triple mutant plants and looked for alterations imposed by hypoxia to the expression of selected differentially expressed genes (DEGs). The five genes (three upregulated and two downregulated) chosen as markers displayed a rather heterogeneous behaviour (Figure 4B). GRX480 maintained hypoxic inducibility irrespective of the genotype, whereas JAZ1 and At2g47950 lost it in one or in both knockout backgrounds, respectively (Figure 4B). Like GRX480, the regulation of each selected repressed gene (MAN7 and NLM2) was unaffected by multiple gene knockout (Figure 4B). Altogether, these results supported a complex picture of JA-mediated gene expression under hypoxia, only partially involving canonical JA or hypoxia signalling and probably requiring additional components.

### 2.5. JA Signaling Is Active under Hypoxia and Contributes to Root Growth Restriction

**Figure 5:**
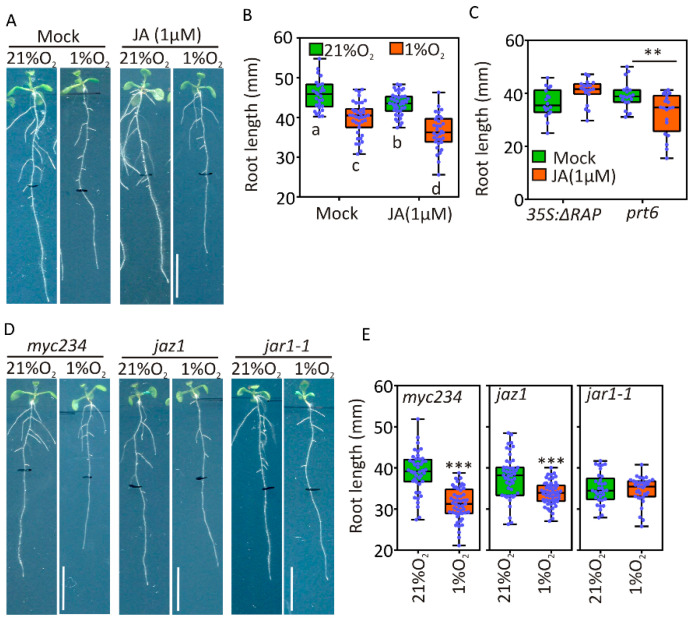
Involvement of JA signalling components in root growth repression under hypoxia: (**A**) Root phenotype of 11-day-old Col-0 plants treated for four days with 1% and 21% O_2_ atmosphere in the presence or absence of 1 µM jasmonic acid for four days (size bar: 10 mm); (**B**) root length of 11-day-old Col-0 plants treated with 1% and 21% O_2_ in the presence or absence of 1 µM JA for four days (*n* ≥ 30) (*p* < 0.05, Two-way ANOVA, Tukey’s post hoc test); (**C**) root length of 11-day-old Δ13RAP and prt6 plants treated with 1 µM JA (*n* ≥ 20) (*p* < 0.05, T-test); (**D**) root phenotype of myc234, jaz1 and jar1-1 11-day-old plants treated for four days in 1% and 21% O_2_ (size bar: 10 mm); and (**E**) root length of 11-day-old myc234, jaz1 and jar1-1 plants treated for four days in 1% and 21% O_2_ (*n* ≥ 30). The wildtype control is presented in 5A and 5B. Asterisks indicate statistical significance compared with untreated controls (**p ≤ 0.01, ***p ≤ 0.001 t-test) (size bar: 10mm).

The observations collected in the previous experiments encouraged us to test the effect of exogenous jasmonates on root growth under aerobic and hypoxic conditions. This could reveal whether the repression of primary root elongation imposed by hypoxia is saturated or can be further enhanced by JA. Root length was significantly reduced in the wildtype by a 4-day-long treatment with hypoxia (1% O_2_ *v*/*v*), as observed before, and further inhibited by the presence of 1 μM JA (Figure 5A,B). A two-way ANOVA analysis did not detect significant interaction between the two treatments. We tested the additional repressive capacity of exogenous JA treatment also in seedings with constitutively active hypoxia signalling. prt6 but not ΔRAP roots showed Ja-sensitivity, although a stronger treatment (5 µM Methyl-JA) was able to repress growth even in the latter genotype (Figure 5C). Together, these observations indicated that ectopic expression of RAP2.12 saturates the repressive effect of moderate JA signalling on primary root growth, although its limited induction in hypoxia and the prt6 background is not sufficient for it. To further study the JA-related signalling cascade involved in this regulatory process, we assessed the extent of root growth repression in JA-insensitive mutants under hypoxia. We observed that primary root growth was reduced in myc234 and jaz1 mutants, and exposure to 4 d hypoxia further inhibited it to a similar extent as in wildtype plants (Figure 5A,B,D,E). Instead, inactivation of the JA-Ile conjugation enzyme JAR1 caused reduced primary root growth under aerobic conditions but did not show further decrease under hypoxia, suggesting that in this genotype active root growth repression by JA signalling was lost (Figure 5D–E). We therefore concluded that hypoxia triggers a JA-mediated signalling pathway that is independent of the release of JAZ-MYC2 repressive interaction.

## 3. Discussion

Throughout their lifespan, plants accumulate a limited amount of resources that can be dedicated to either growth and reproduction or enduring biotic and abiotic stresses. Therefore, when plants face adverse environmental conditions, growth and development are necessarily affected [4,5]. In the frame of this study, we focused on the inhibition of primary root elongation that occurs under hypoxia. We confirmed that elongation of the primary root of Arabidopsis plants grown on agarised plates was significantly hampered by imposition of a hypoxic atmosphere (Figure 1A,D,F). The slanting tropism reported by Eysholdt-Derzso and Sauter [54] could still be observed, although to a moderate extent (Figure 1A). This can be explained by the fact that, differently from the original report of this phenomenon, hypoxic treatments were applied to plants maintained under photoperiodic regime in order to avoid interference with light signalling and possible starvation due to prolonged darkness. Therefore, the photo-oxygenic activity likely raised the oxygen concentration inside the plate when oxygen was exchanged with the external atmosphere through the stomata. Nevertheless, continuous flushing of an atmosphere containing 1% O_2_ was sufficient to restrict root elongation (Figure 1A,B).

Remarkably, primary root growth was maintained at a reduced rate four days after the end of the hypoxic treatment (Figure 1C, Supplementary Figure S1), which led us to speculate about the persistence of an inhibiting signalling after reoxygenation. It should be noted, however, that Arabidopsis root elongation in vertical plates is expected to increase with plant age, as reported by Yazdanbakhsh and Fisahn [55]. The comparable growth rate observed for seedlings grown permanently under aerobic conditions for 11 days and 15-day-old ones treated for 4 days with hypoxia (Supplementary Figure S1) can be considered as a simple delay imposed by active repression under hypoxia and fully relieved by restoration of aerobiosis.

The involvement of JA signalling in the active restriction of root growth that arose from the current study (Figure 5) is rather controversial, considering the requirement of several oxygen molecules for JA biosynthesis [56]. However, it is possible that, under naturally occurring hypoxia, such as waterlogging, the actual oxygen available in the non-submerged organs sustains JA biosynthesis, which is subsequently transported basipetally to restrict root meristem activity. Indeed, we could only observe significant increase of the Ile-conjugated form of JA, whereas the unconjugated version or cis-OPDA precursor were unaffected (Figure 3), suggesting that it is the last step of JA activation to be stimulated by short-term hypoxia. Recently, JA influx and efflux transporters have been identified in Arabidopsis [57]. The transient nature of Ja-Ile accumulation under hypoxia could thus also be likely explained by the energy requirement of AtABCG16/jasmonate transporter 1 for its activity [58]. Moreover, the observation of considerably lower levels of cis-OPDA over prolonged hypoxia when compared to aerobic roots suggested that, in the long run, oxygen and ATP became limiting factors in the synthesis of this hormone. In addition to substrate shortage, it is also possible that the JA biosynthetic pathway is actively repressed at the transcriptional or posttranscriptional levels by hypoxia. For instance, a reduction in JA-biosynthetic gene expression and consequently of JA levels in the seedlings of the N-end rule pathway mutant of Arabidopsis ate1ate2 has been reported previously [59]. Additionally, moderate repression of the JA-biosynthetic genes AOC2, OAS, VSP1 and JAR1 by hypoxia was also observed in root cell types of Arabidopsis [32].

The role of JA in root growth inhibition has been extensively characterized in the past (reviewed in [60]). This regulatory activity has been shown to be mediated by different transcriptional regulators belonging to the basic helix-loop-helix (bHLH) and ethylene insensitive 3 (EIN3) families [61]. The observed root inhibition under hypoxia does not seem to be mediated by the bHLH MYC2, 3 and 4, as indicated by maintenance of repression in a myc234 mutant (Figure 5). In fact, whereas MYC2 has been shown to repress PLT1 and PLT2, thereby inhibiting room meristem activity [43], hypoxia and possibly its JA-signalling component seem to act post-transcriptionally on PLT2 and PLT3 (Figure 2). Transcriptional repression was instead observed for WOX5, which therefore suggests a parallel regulatory pathway to be in action (Figure 2D). Since significant, although slight, WOX5 repression could be measured in a ΔRAP genotype (Figure 2A), it is tempting to speculate about a repressive role for the ERF-VII transcription factors in the root apical meristem. Further analyses in plants impaired in jasmonate synthesis or signalling will be required to test this hypothesis. Their involvement seems to be indirect, since we could not identify the HRPE motif, preferentially recognized by ERF-VII, in the promoters of most JA responsive genes (Supplementary Table S4). This role might rather be ascribed to transcriptional repressors activated by ERF-VIIs under hypoxia, such as the Hypoxia Responsive Attenuator 1 or Lateral Organ Boundaries (LOB) domain-containing protein 41 [10,62]. Further analyses are therefore required to shed light on the role of these transcription factors in limiting root growth under low oxygen environment.

In summary, with this study, we provided novel evidence that, under hypoxic conditions, restriction of root growth relies at least partly on JA-signalling pathways that negatively affect PLT2/3 and WOX5 activity. The synthesis of jasmonate is initially enhanced under such stress in the root, although it later decreases, probably due to oxygen and energy deficiency. We also provided the first evidence that the main known mechanism accounting for plant transcriptional responses to low oxygen, which is based on ERF-VII stabilisation, might be intertwined with this process. These observations will need to be corroborated in the future with similar analyses using single and high-order level erf-VII mutants. This study paves the way to understanding adaptive root growth responses under localised hypoxic conditions and therefore suggests previously unrecognized targets for breeding crops with improved waterlogging tolerance.

## 4. Materials and Methods

### 4.1. Plant Material and Growth Conditions

The Columbia-0 (Col-0) ecotype of *Arabidopsis thaliana* was used as the wildtype background in all experiments. The transgenic line expressing a stabilised RAP2.12 factor (35S:∆13RAP #10) used for experiments has been previously described in [12]. The ERF-VII mutant [50] was provided by Michael Holdsworth (University of Nottingham, UK). *prt6-5* (N684039), *jar1-1* (N8072) and *jaz1* (N2107784) seeds were obtained from the European Arabidopsis Stock Centre (NASC). myc234 mutant seeds were previously described in [63] and provided by Roberto Solano (Spanish National Research Council, Madrid, Spain). Plethora reporter lines, pPLT1:PLT1-YFP, pPLT2:PLT2-YFP, pPLT3:PLT3-YFP, and pPLT4:PLT4-YFP and WOX5 reporter line WOX5:GFP, were previously described in [64] and provided by Ben Scheres (University of Wageningen). A jasmonate responsive reporter, Jas9-VENUS, has been previously described in [65] and was provided by Laurent Laplaze (Institute of Research for Development, Marseille). Seeds were grown on vertical plates on agarised medium composed of half-strength Murashige and Skoog (Duchefa) basal salt mixture, 0.8% plant agar, supplemented with 1% sucrose (Sigma-Aldrich). Seeds were stratified at 4 °C in the dark for 48 h and germinated at 22 °C day/18 °C night with a photoperiod of 12 h light and 12 h dark. For root development evaluation, treatments were applied as described in [31] to plants grown in air containing 21% oxygen (normoxia) or 1% O_2_ as hypoxia for the time indicated in the figure legends and while keeping plants under unchanged growth photoperiod.

### 4.2. Measurement of Primary Root Length

Transparent square plates (12 cm side) containing the Arabidopsis plants were scanned at the end of the treatments, using a Perfection V700 Photo scanner (Seiko Epson) at the resolution of 300 DPI. The software EZ–Rhizo [66] was used for root length measurement, while the number of emerged lateral roots was calculated manually.

### 4.3. Confocal Imaging

For GFP and YFP visualisation in the primary root, plants were grown for eleven days on vertical square plates. Roots were observed under a FluoView1000 (Olympus) inverted confocal laser scanning microscope with a 20× objective lens. GFP fluorescence was excited with 488-nm laser light (4% laser transmissivity, PMT voltage 805 V) and collected with a 497–554 nm long-pass emission filter. YFP fluorescence was excited with 515-nm laser light (10% laser transmissivity, PMT voltage 890 V) and collected with a 520–560 nm long-pass emission filter. Propidium iodide (PI) fluorescence was excited with 515-nm laser light (10% laser transmissivity, PMT voltage 905 V) and collected at 590–680 nm. Scanner and detector settings were optimized and kept unchanged for all the experiments. Images were analysed with the FluoView FV1000 software (Olympus).

### 4.4. In Silico Analysis of Differentially Expressed Genes

Differentially expressed genes (DEGs) upon jasmonate treatment were selected from the microarray experiment by [67] (−0.5 < log2FC ≥ 2, corresponding to 70 DEGs in that data set) and compared to the hypoxic root microarray profiles produced by [32], as retrieved from the Genevestigator platform [65]. JA-responsive genes for which mRNA association with ribosome and/or overall abundance is affected by hypoxia were selected by filtering those coherently up- or downregulated in at least one root cell-type.

### 4.5. RNA Extraction and Gene Expression Analysis by Real-Time qRT-PCR

Roots from 11-day-old Arabidopsis plants grown on vertical agar plates were collected and immediately frozen in liquid nitrogen. RNA extraction was performed and processed to cDNA as described in [68]. An ABI Prism 7300 sequence detection system (Applied Biosystems) was used to carry out real-time PCR, using iQSYBR Green Supermix (Biorad). Ubiquitin10 (At4g05320) was used as the housekeeping gene. Relative expression of each gene was calculated using the 2^-∆∆Ct^ method [69]. A full list of the qPCR primers used is provided in Supplementary Table S1.

### 4.6. Hormone Quantification

Quantification of JA, cis-OPDA and JA-Ile was performed according to the method described in [70]. Samples were homogenized, and hormones were extracted using an aqueous solution of 10% MeOH/H_2_O, *v*/*v*. The extracts were purified using Oasis HLB columns (30 mg/L mL, Waters), and hormones were eluted with 80% MeOH. Eluent was evaporated to dryness under a stream of nitrogen. Jasmonates levels were determined by ultra-high performance liquid chromatography-electrospray tandem mass spectrometry (UHPLC-MS/MS) using Acquity UPLC^®^ System (Waters) equipped with an Acquity UPLC BEH C18 column (100 × 2.1 mm, 1.7 μm; Waters) coupled to triple quadrupole mass spectrometer Xevo™ TQ-S MS (Waters).

### 4.7. Statistical Analysis

Sigmaplot 14 (Systat Software) was used to carry out statistical analyses. Comparisons between treatments and genotypes were performed, after testing the normality of data distribution, using t-test or Analysis of Variance (ANOVA) depending on the number of averages. In the case of ANOVA, a Tukey post hoc test was applied for pairwise comparison. Results shown in figures are representative of those obtained from experiments repeated twice with qualitatively equal relative output.

## Supplementary Materials

The following are available online at https://www.mdpi.com/2223-7747/9/8/1046/s1. Figure S1. Primary root growth rate in aerobic, hypoxic and post-hypoxic recovery conditions. Figure S2. Effect of hypoxia on PLT1 and PLT4 protein level in the root apex. Figure S3. Effect of hypoxia on the expression JAZ genes in the roots of wildtype and oxygen or jasmonate sensing mutants. Figure S4. Effect of 5 µM methyl-jasmonate (MeJA) treatment on Δ13RAP root growth. Table S1. List of oligonucleotides used for real-time qPCR in this study. Table S2. Expression levels of up- or downregulated genes (log fold change |1|) in response to Me-JA treatments [51] and the corresponding fold change in ribosome association caused by short-term exposure to hypoxia in root tissues [32]. Table S3. Numerical values of the expression levels of 24 JA-responsive genes shown as a heatmap in Figure 4. Table S4. Presence of HRPE motif in the promoter of JA-regulated genes shown in Figure 3.
