# Supplementary material for: Jasmonate Signalling Contributes to Primary Root Inhibition Upon Oxygen Deficiency in Arabidopsis thaliana
Source: Plants (Basel). 2020 Aug 17;9(8):1046. doi: 10.3390/plants9081046 (PMC7464498; doi:10.3390/plants9081046)
Supplement: Supplementary file 1 [file plants-09-01046-s001.zip › plants-852519-SI/Supplementary Materials.pdf]

**Supplementary Figures and Tables**

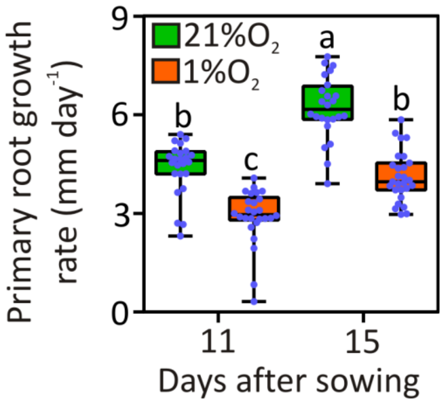


**Supplementary Figure S1**. Primary root growth rate in aerobic, hypoxic and post-hypoxic recovery conditions. Root elongation of 7 d old wild-type Arabidopsis plants grown under aerobic (green, 21% $O_2$) and hypoxic (orange, 1% $O_2$) conditions for four days and subsequently allowed to recover for four days under aerobic conditions. The growth rate is calculated as the difference between the root length measured for each plant at the time point shown on the x axis and four days before (n ≥ 20). Letters are used to distinguish statistically different groups (2-way ANOVA, Tukey post-hoc test, $p \leq 0.05$).

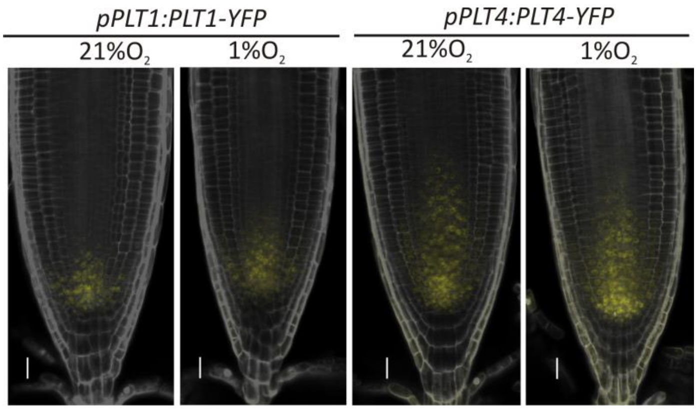


**Supplementary Figure S2**. Effect of hypoxia on PLT1 and PLT4 protein level in the root apex. YFP signal in the root apex of *pPLT1:PLT1-YFP* and *pPLT4:PLT4-YFP* plants maintained 12 hours under aerobic (21% $O_2$) and hypoxic (1% $O_2$) conditions. Size bar = 20 μm.

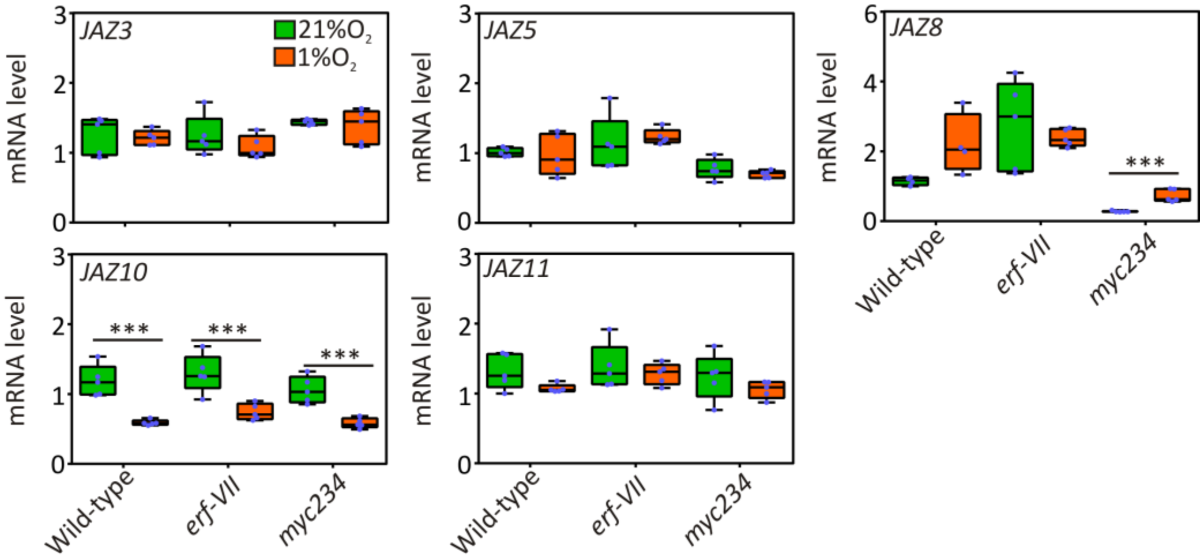


**Supplementary Figure S3.** Effect of hypoxia on the expression JAZ genes in the roots of wild-type and oxygen or jasmonate sensing mutants. Expression of five selected JAZ genes in wild-type, *erf-VII* and *myc234* mutants, under aerobic (green, 21% $O_2$) and hypoxic (orange, 1% $O_2$) conditions. mRNA level of each gene is normalized to the house-keeping gene (UBQ10-AT4G05320) to calculate ΔΔCt. Data shown in the graphs are the re-normalized ΔΔCt values to one of the biological replicate of Wild-type sample at 21% $O_2$. Data are mean ± SD of five biological replicates. Asterisks mark statistically significant differences in the indicated pairwise comparisons (*** $p \leq 0.05$, t- test).

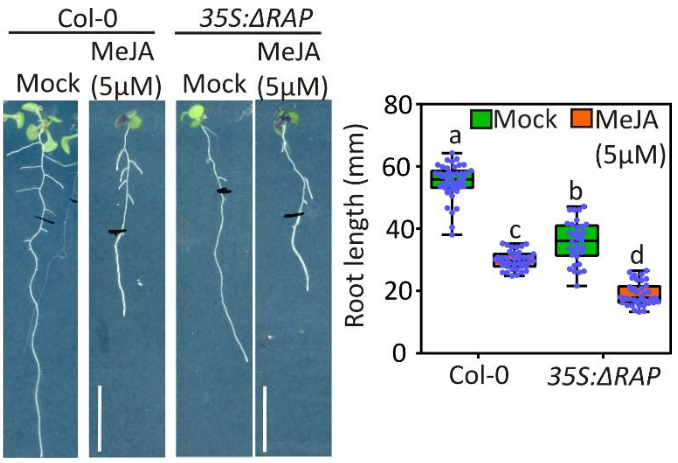


**Supplementary Figure S4.** Effect of 5 µM methyl-jasmonate (MeJA) on Δ13RAP root growth. (Left) Root phenotype of 11 d old Δ13RAP plants treated for four days with 5 µM methyl jasmonate (Size bar = 10 mm). (Right) Root length of 11 d old Δ13RAP plants treated with 5 µM methyl jasmonate ($n \geq 30$). (2-way ANOVA, Tukey post-hoc test, $p \leq 0.05$).

**Supplementary Table S1.** List of oligonucleotides used for realtime qPCR in this study.

| qPCR primers | | |
|---|---|---|
| **Primer name** | **AGI code** | **Primer sequence (5'->3')** |
| sgGRX480_fw | *AT1G28480* | ACGGAGAGGATGTTGCATGTGTC |
| sgGRX480_rv | | AATCTCAAGGACCGCCGGATTC |
| sgAT2G47950_Fw | *AT2G47950* | AAATCTGGCTGTGGATTCTGCTG |
| sgAT2G47950_Rv | | TGCGAGACAAGCTTCCCAGAAC |
| sgMAN7_fw | *AT5G66460* | AATCCTTGGAGATGGCAACAGC |
| sgMAN7_rv | | TCTGAACCAGCACATAGCCTTGG |
| sgNIP1-2_fw | *AT4G18910* | GCCGTTTCCCTCTTAAACAGGTTC |
| sgNIP1-2_rv | | CCGAACAAGAGCCGTAAAGTCG |
| sgPLT1_fw | *AT3G20840* | AGTTCGTGGCTGCCATTAGAAGG |
| sgPLT1_rv | | ATCTTCCATGTTGGTGATGCCTTG |
| sgPLT2-F | *AT1G51190* | GAAGATGGCAAGCAAGGATCGG |
| sgPLT2-R | | GCTTCTTCCTCCGTGCTGAATG |
| sgPLT3-F | *AT5G10510* | GCTGTTGTGGCTGTGGAAACATC |
| sgPLT3-R | | TCTTCCCGTCCATCTATGTCGAG |
| sgPLT4-F | *AT5G17430* | ACCCTCGAAGCTTTCACCAGAG |
| sgPLT4-R | | ACCACCATTGATGTCCCAATCTCG |
| sgWOX5_fw | *At3g11260* | CGTGAAAGGTCGAAGCTTACGTG |
| sgWOX5_rv | | ATTCCATCTCCCGCACTTCGTC |
| sgJAZ1_Fw | *AT1G19180* | AGCTTCACTTCACCGGTTCTTGGA |
| sgJAZ1_rv | | TCTTGTCTTGAAGCAACGTCGTCA |
| sgJAZ3_Fw | *AT3G17860* | CTGCCCAGTACCAGTTTCTTGTTC |
| sgJAZ3_rv | | AGCCAGAGGTAATGCCACTGTTG |
| sgJAZ5_Fw | *AT1G17380* | TTCCAAAGGCGAACCCTCTACC |
| sgJAZ5_rv | | TCCTGGCTGTGATTCACTGAGG |
| sgJAZ8_Fw | *AT1G30135* | GGTCGGATCCTCCAAACAAGTC |
| sgJAZ8_rv | | TCGTCGTGAATGGTACGGTGAAG |
| sgJAZ10_fw | *At5g13220* | TCGAGAAGCGCAAGGAGAGATTAG |
| sgJAZ10_rv | | TCGTTTAGGCCGATGTCGGATAG |
| sgJAZ11_fw | *At3g43440* | TGCCAATTGCTAGGAGACGTTCAC |
| sgJAZ11_rv | | GTAAGGCTTCGTGTGGACAAATCG |

**Supplementary Table S4.** Presence of HRPE motif in the promoter of JA-regulated genes shown in Fig. 4. The 5'-AAACCA(G/C)(G/C)(G/C)GC-3' element was searched in 1 kb upstream of the translation start site (ATG) of each gene. Sequences that displayed such feature are highlighted in bold. The position of the identified element is relative to the ATG site.

| | Gene Symbol | AGI code | HRPE element start | stop | strand | score | p-value |
|---|---|---|---|---|---|---|---|
| Up-regulated genes | | *AT5G14730* | NA | NA | NA | NA | NA |
| | | *AT1G76600* | NA | NA | NA | NA | NA |
| | *GRXC9* | *AT1G28480* | NA | NA | NA | NA | NA |
| | *WRKY40* | *AT1G80840* | NA | NA | NA | NA | NA |
| | *BCB* | *AT5G20230* | NA | NA | NA | NA | NA |
| | *NAC102* | *AT5G63790* | NA | NA | NA | NA | NA |
| | | *AT2G47950* | NA | NA | NA | NA | NA |
| | | ***AT5G12340*** | 185 | 196 | + | 14.2727 | 6.11E-06 |
| | | ***AT5G19110*** | 32 | 43 | + | 10.2222 | 8.88E-05 |
| | *TIFY10A* | *AT1G19180* | NA | NA | NA | NA | NA |
| | *UGT73B4* | *AT2G15490* | NA | NA | NA | NA | NA |
| Down-regulated genes | *BRL2* | *AT2G01950* | NA | NA | NA | NA | NA |
| | *MAN7* | *AT5G66460* | NA | NA | NA | NA | NA |
| | *CEL1* | *AT1G70710* | NA | NA | NA | NA | NA |
| | *NIP1-2* | *AT4G18910* | NA | NA | NA | NA | NA |
| | *SMO1-1* | *AT4G12110* | NA | NA | NA | NA | NA |
| | *LAX2* | *AT2G21050* | NA | NA | NA | NA | NA |
| | | *AT4G09890* | NA | NA | NA | NA | NA |
| | | *AT4G39320* | NA | NA | NA | NA | NA |
| | *CYP90B1* | *AT3G50660* | NA | NA | NA | NA | NA |
| | | *AT3G10080* | NA | NA | NA | NA | NA |
| | | ***AT1G04680*** | 130 | 141 | + | 10.2727 | 8.63E-05 |
| | | *AT5G51560* | NA | NA | NA | NA | NA |
| | | *AT4G35720* | NA | NA | NA | NA | NA |
